# Five-Year Prospective Study on Implant Failure and Marginal Bone Remodeling Expected Using Bone Level Implants with Sandblasted/Acid-Etched Surface and Conical Connection

**DOI:** 10.1055/s-0041-1739439

**Published:** 2022-01-06

**Authors:** Marco Tallarico, Aurea Maria Immacolata Lumbau, Silvio Mario Meloni, Irene Ieria, Chang-Joo Park, Lukasz Zadrożny, Erta Xhanari, Milena Pisano

**Affiliations:** 1 School of Dentistry, University of Sassari, Sassari, Italy; 2 Private Practice, Rome, Italy; 3 Department of Dentistry, Division of Oral and Maxillofacial Surgery, College of Medicine, Hanyang University, Seoul, Korea; 4 Department of Dental Propedeutics and Prophylaxis, Medical University of Warsaw, Warsaw, Poland; 5 Department of Implantology and Prosthetic Aspects, Master of Science in Dentistry Program, Aldent University, Tirana, Albania

**Keywords:** sandblasted, acid-etched, connection, marginal bone remodeling, marginal bone loss

## Abstract

**Objective** The purpose of the present prospective, case-series study was to report implant survival rate and marginal bone remodeling expected 5 years after loading using dental implants placed in daily practice.

**Materials and Methods** This research was designed as an open-cohort, prospective, case-series evaluation. Any partially or completely edentulous patient, scheduled to receive at least one bone level implant, was considered eligible for this study. Primary outcome measurements were: implant and prosthetic cumulative survival rate and any complications experienced up to the 5-year follow-up. Secondary outcome measures were: thickness of gingival biotype, implant insertion torque, implant stability quotient, and marginal bone loss (MBL).

**Results** Ninety consecutive patients (34 males and 56 females, aged between 24 and 81 years old [mean: 53.2 ± 15.4]) with 243 inserted implants were followed for at least 5 years after loading (mean: 65.4 ± 3.1 months; range from 60 to 72). At the 1-year follow-up, no drop-outs were recorded, but 17 patients (18.9%) with 18 restorations (12.6%) delivered on 34 implants (14%) were lost at the 5-year examination. At the 5-year follow-up examination, six implants lost osseointegration (97.5%). In the same period, four prostheses failed (97.2%). Five complications were reported in five different patients (prosthetic success rate was 96.5%, at patient level). Five years after loading, the mean MBL was 0.41 ± 0.30 mm. The difference from the 1-year data was 0.04 ± 0.19 mm. A statistically significant higher MBL was found for smokers, and patients with thin gingival biotype. The mean implant insertion torque was 42.9 ± 4.8 Ncm (range from 15 to 45 Ncm). Two-hundred and three implants (83.5%) were inserted with an insertion torque ≥35 and ≤45 Ncm.

**Conclusions** High implant survival and success rate could be expected with stable marginal bone remodeling up to 5 years after loading. Smoking and thin tissue biotype were the most important variabilities associated with higher MBL. Further research studies are needed to confirm these results.

## Introduction

Bone remodeling around dental implants at early stages is one of the most critical factors in predicting implant success. In the past, it was believed that a physiological marginal bone loss (MBL) of 1.5 to 2.0 mm was expected around a dental implant during the first year of function. 1 After that, a minimal bone loss would be observed. 2 3 4 Several factors may increase the physiological MBL, including but not limited to the biological width establishment, surgical trauma, implant–abutment connection type, soft tissue thickness and quality, and implant design. 5 6 7 8 To make the situation even more complex, several pathological co-factors, including genetic predisposition, history of periodontitis, smoking, diabetes, poor plaque control, as well as, some iatrogenic factors, may contribute to increase peri-implant bone loss. 9 10 11 12 13

Modern implantology changed the way to define implant success. Papaspyridakos and coworkers 4 proposed some parameters related to the soft- and hard-tissue stability around implants. Later, Galindo-Moreno and coworkers 8 demonstrated that implants with increased physiological MBL may compromise their final outcomes. Therefore, MBL of more than 0.44 mm/year is a strong indication of peri-implant bone loss progression. In 2013, the American Academy of Periodontology defined the “peri-implantitis” as an “ *inflammatory reaction associated with the loss of supporting bone beyond the initial biological bone remodeling around an implant in function* .” 14 Finally, Tallarico and coworkers proposed, as a part of a consensus conference on peri-implantitis, an etiology-driven classification to assist the clinician in detecting and classifying the etiology-based peri-implantitis. 15 However, there is still confusion whether the physiological and pathological bone remodeling are host-related, prosthesis-, and/or implant-related, as well as load-dependent. 4

To maintain the physiological marginal bone remodeling as lower as possible, clinicians should be well aware of the biological and mechanical process occurring at the implant–abutment connection, as well as the features of used implants. This is mandatory to understand the expected physiological marginal bone remodeling and any relationship between explanatory variables and pathological MBL, preventing early and further implant failures.

The purpose of the present prospective, case-series evaluation was to analyze survival and success rates of implant-supported restoration placed in the daily practice, as well as the marginal bone remodeling expected after implant placement, and up to 5 years after loading. The intent was to understand possible variabilities associated with implant failure and peri-implantitis. This study was written according to the STROBE statement.

## Materials and Methods

This research represents the 5-year follow-up of a previous preliminary report. 16 Originally, this study was designed as an open-cohort, prospective case-series evaluation. Surgical and prosthetic treatments were performed from September 2014 to December 2016, by a certified clinician (M.T.). Enrolled patients were treated consecutively, as a part of routine treatments, once their written consent had been obtained. Patients were informed about the nature of the study, including clinical procedures, materials, benefits, potential risks, and complications of the proposed treatments. This study was conducted according to the principles embodied in the Helsinki Declaration of 1975, as revised in 2008. The publication of the present research was approved by the Ethical Committee of Aldent University, Tirana, Albania (2/2021).

Any partially or completely edentulous patient who was scheduled to receive at least one bone level implant (Osstem TSIII, Osstem Implant Co. Ltd., Seoul, South Korea), featured with a sandblasted and acid-etched surface (rough surface [Ra] of 2.5–3.0 μm), and internal conical connection of 11°, was considered eligible for this study. As this research was designed as an open-cohort prospective evaluation, any implant and prosthetic location/design and any surgical and loading protocol were considered. Exclusion criteria are reported in Table 1 .

**Table 1:** Exclusion criteria

American Society of Anesthesiologists class III and IV
Patients under treatment or treated in the past 5 years with intravenous amino-bisphosphonates
Radiotherapy of the oral and maxillofacial region (<5 years)
Uncontrolled periodontal disease (bleeding on probing [BoP] and/or plaque index [PI] ≥ 25%)

Initial screening and case evaluation were performed as shown in Table 2 .

**Table 2:** Steps of the initial screening evaluation

Medical and dental records
Needs and expectations of patients
Comprehensive periodontal evaluation
Periapical radiographs, panoramic radiographs, or cone beam computed tomography (CBCT)
Preoperative photographs
Digital or conventional study models

## Surgical and Prosthetic Protocols

Complete surgical and prosthetic procedures were reported in the previous publication. 16 In brief, patients received antibiotic (2 g of amoxicillin or 600 mg of clindamycin if allergic to penicillin) 1 hour before surgery. Implants (Osstem TSIII, Osstem Implant Co. Ltd.) were placed at the bone level or slightly below using either conventional freehand surgery or computer-guided/template-assisted implant placement. In case of immediate postextractive implants, fixtures were placed 1.5 mm below the buccal bone plate. All the implants were placed following the drilling protocol recommended by the manufacturer. A flapless approach was planned in the case of postextractive implants or in a healed site, according to the width of the available keratinized mucosa. In cases of ridge atrophy (bone height < 7.0 mm and/or bone width < 4.5 mm), implant placement was performed simultaneously to guided bone regeneration (GBR). Nevertheless, in cases of severe ridge atrophy, including damage of the residual alveolar socket, implant placement was performed 4 to 6 months after bone regeneration/socket preservation procedures. Sinus lift was performed using the lateral approach in case of residual bone height lower than 3 mm, or by a less invasive transcrestal sinus floor elevation (Crestal Approach Sinus KIT, CAS-KIT, Osstem Implant Co. Ltd.), in case the residual alveolar bone crest was at least 3 mm, as measured on pre-operative CBCT scan. The loading protocol was initially planned on individual case requirements, but finally performed according to the primary implant stability. Hence, one-stage approach and immediate loading were performed with a primary implant stability of at least 35 Ncm. In case of immediate loading, prefabricated restorations were trimmed and polished chair-side, and delivered in the same surgical session. Nonoccluding, temporary restorations were delivered in partially edentulous patients, while, complete edentulous patients received splinted, metal-reinforced, temporary restorations with centric contact and group function, without any cantilever. All of the patients received oral and written recommendations on medication, oral hygiene maintenance, and diet. In case of immediate implants, bone regeneration, and/or sinus procedures, postoperative antibiotic therapy (1 g of amoxicillin or 300 mg of clindamycin) was continued every 12 hours for 6 to 8 days. Analgesics were administered as needed.

**Fig. 1:**
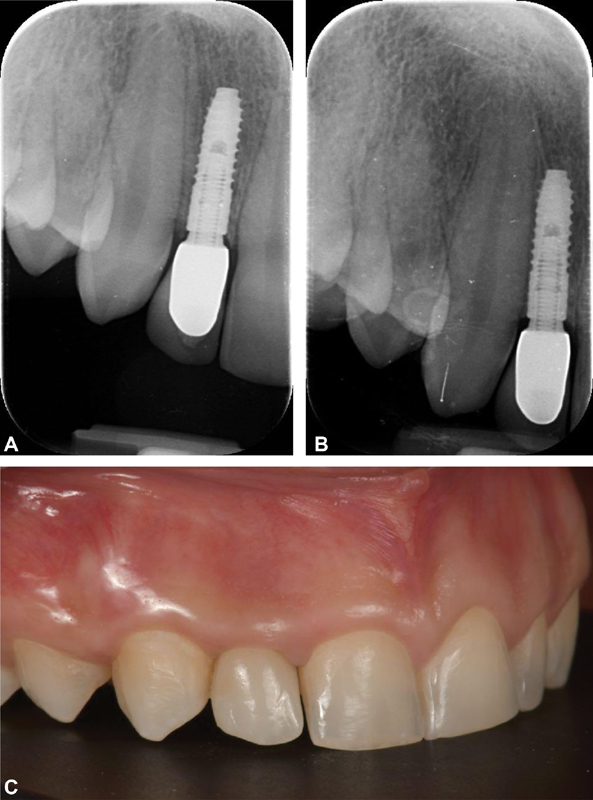
( **A** ) Case 1 (narrow implant): periapical radiograph at the definitive prosthesis delivery. ( **B** ) Case 1: periapical radiograph at the 5-year follow-up. ( **c** ) Case 1: intraoral picture at the 5-year follow-up.

**Fig. 2:**
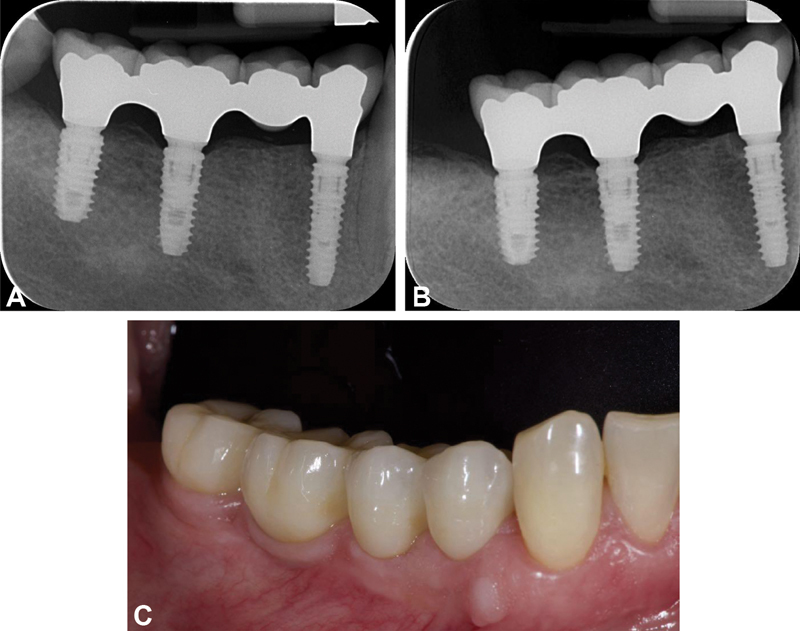
( **A** ) Case 2 (fixed partial restoration on regal implants): periapical radiograph at the definitive prosthesis delivery. ( **B** ) Case 2: periapical radiograph at the 5-year follow-up. ( **c** ) Case 2: intraoral picture at the 5-year follow-up.

**Fig. 3:**
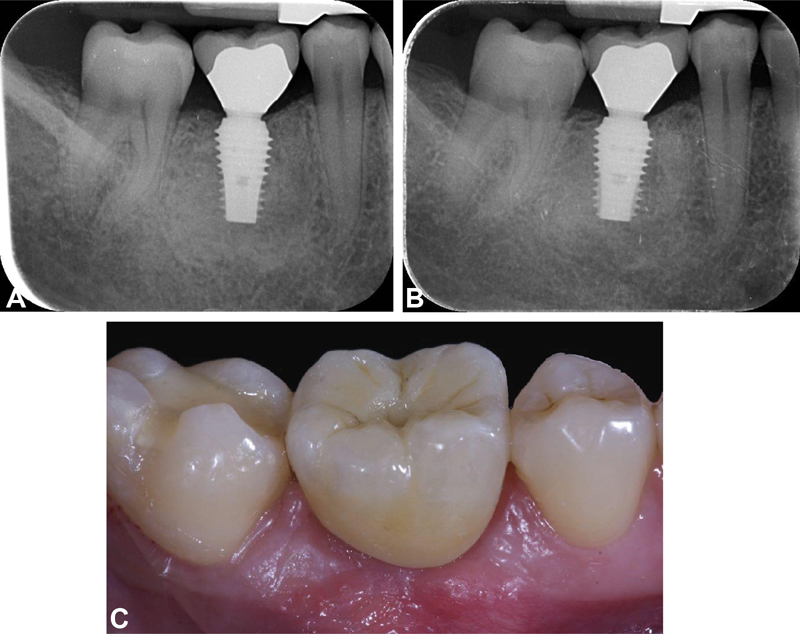
( **A** ) Case 3 (wide diameter implant): periapical radiograph at the definitive prosthesis delivery. ( **B** ) Case 3: periapical radiograph at the 5-year follow-up. ( **C** ) Case 3: intraoral picture at the 5-year follow-up.

**Fig. 4:**
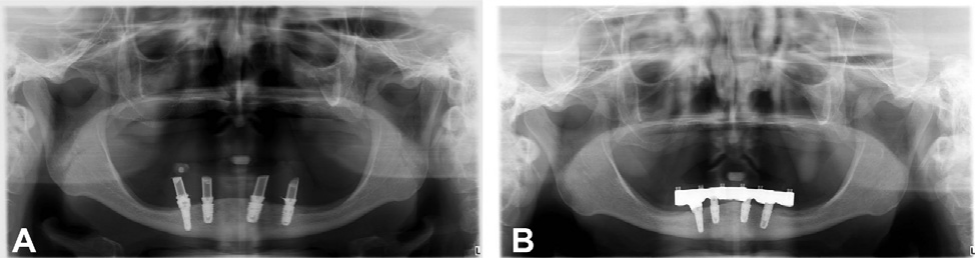
( **A** ) Case 4 (complete-arch restoration): panoramic radiograph at the definitive prosthesis delivery. ( **B** ) Case 4: panoramic radiograph at the 5-year follow-up.

**Fig. 5:**
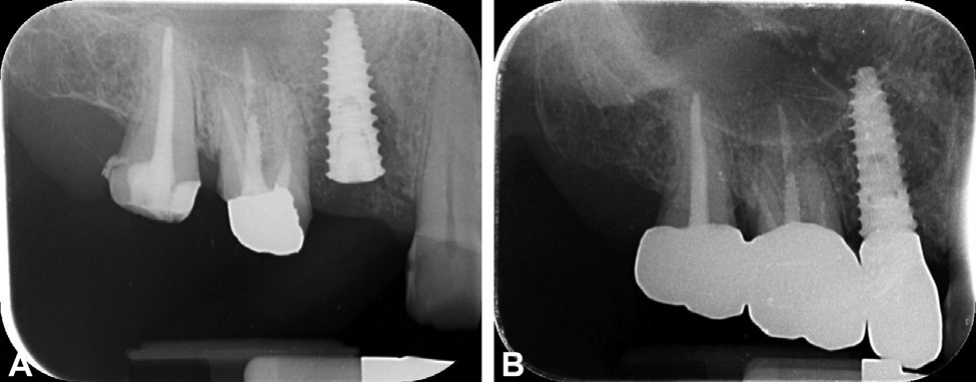
( **A** ) Case 5 (biological complication): periapical radiograph 1 month after implant placement. ( **B** ) Case 5: periapical radiograph at the 5-year follow-up.

Overdentures and definitive single and partial crowns were delivered 8 weeks after implant placement, according to an early loading protocol; complete arch restorations were delivered after 20 weeks. In case of bone augmentation procedures, or immediate implants, definitive restorations were delivered 4 to 6 months after second-stage or initial loading, respectively. Definitive restorations were either cemented or screw-retained, delivered on either stock or customized computer-assisted design/computer-assisted manufacturing (CAD/CAM) abutments. Multi-abutments (Osstem Implant Co. Ltd.) or OT Equator (Rhein83, Bologna, Italy) were used as intermediate abutments, in case of complete arch restorations. After definitive prosthesis delivery, all the patients were scheduled for a standard hygiene recall program. Periapical radiographs were taken after definitive prosthesis delivery and then annually. Occlusion was checked and adjusted at each recall appointment. Explanatory cases are illustrated in Figs. 1 to 5 .

## Outcome Measures

Primary outcome measures were the success rates of implants and prostheses, and any complications experienced during the entire follow-up period. Outcomes were assessed by two operators (E.X. and I.I.), both not previously involved in this research, at 1- (E.X.) and 5-year (I.I.) follow-up examinations, respectively. Implant failure was defined as mobility assessed by tapping or rocking the implant head with the metallic handles of two instruments, progressive MBL or infection, and any complications rendering the implant unusable, although still mechanically stable in the bone (for example, implant fracture). Prosthesis failure was defined if it needed to be replaced with another prosthesis. Any biological (pain, swelling, suppuration, etc.) and/or mechanical (screw loosening, fracture of the framework, and/or the veneering material) complications were considered.

Secondary outcome measures were marginal bone levels, insertion torque, implant stability quotient (ISQ), residual alveolar bone quality, and soft tissue thickness.

- Marginal bone levels were evaluated at implant placement (baseline), second-stage surgery, definitive crown delivery, and at 1- and 5-year after loading examinations, by using intraoral digital periapical radiographs taken with a paralleling technique. Radiographs were evaluated using an image analysis software (DfW 2.8, SOREDEX) calibrated at each measure, using the known implant's diameter or length. The distance between the implant platform and the most coronal bone to implant contact was recorded at both mesial and distal margins. The mean value was used in the statistical analyses.
- Insertion torque was recorded at implant placement using the surgical unit. The surgeon (M.T.) evaluated and recorded the values.
–SQs were measured by the surgeon (M.T.) using a resonance frequency analysis device (Osstell Mentor device, Osstell, Gothenburg, Sweden) at implant placement and before definitive restoration delivery. The same clinician who performed surgical and prosthetic procedures (M.T.) recorded the ISQ values.
- Residual alveolar bone quality was assessed directly during the implant-site preparation by the surgeon (M.T.) and reported according to the Lekholm and Zarb classification.
- Soft tissue thickness was recorded at the time of surgery (M.T.), measuring the thickness of the gingiva with a periodontal probe. Soft tissues were considered thin if it measured ≤1 mm and thick if it was >1 mm.

### Statistical Analysis

All the data were collected and recorded in an MS Excel file. A statistician with expertise in dentistry and not previously involved in the study analyzed the data and performed all of the analyses (SPSS V.26; IBM, Chicago, Illinois, United States.). Continuous variables were reported as mean ± standard deviation or median and 95% confidence interval (CI). Ordinal and dichotomous variables were given as percentage. Implants and restorations were the considered statistical units of the analyses. Differences in the proportion of dichotomous outcomes (implant and prosthetic failure, and complications) were compared using the Fisher's exact test. Differences in mean for continuous outcomes (MBL and ISQ) were compared by independent samples *t* -test and one-way analysis of variance, respectively. Comparisons between time points and baseline were made by unpaired *t* -tests. Statistical analyses were conducted at the 0.05 level of significance.

## Results

A total of 92 patients were enrolled for this research. Of these, only two patients were excluded (patients refused to participate). Finally 90 consecutive patients (34 males and 56 females; mean age: 53.2 ± 15.4 years old; range from 24 to 81) were definitively treated and data analyzed. Overall, 243 implants were placed and followed up for at least 5 years after loading (mean of 65.4 ± 3.1 months; range from 60 to 72). Two-hundred and eight implants were placed in nonsmoking patients; 20 implants in patients who smoked ≤10 cigarettes/day; and 15 implants in patients who smoked >10 cigarettes/day. The main implant characteristics and distribution are shown in Tables 3 to 5 .

**Table 3:** Main implant characteristics (length and diameter)

Implant length (mm) and diameter (mm)	7.0	8.5	10.0	11.5	13.0	Total
3.0	–	–	–	–	4	4
3.5		2	6	27	10	45
4.0	3	2	17	31	14	67
4.5	3	8	18	8	20	57
5.0	–	1	20	9	–	30
6.0	–	2	11	3	–	16
7.0	–	4	15	5	–	24
Total	6	19	87	83	48	243

**Table 4:** Implant distribution part I

	Central incisors	Lateral incisors	Canines	Premolars	Molars	Total
Maxilla	26	7	4	45	41	123
Mandible	–	15	5	42	58	120
Total	6	19	87	83	48	243

**Table 5:** Implant distribution part II

Implant placement	Immediate implants	43	
	12–16 weeks after tooth extraction and socket preservation	75	
	>4 months after tooth extraction	125	
	Total		243
Loading time	Immediate loading	49	
Guided	Guided implant placement	76	
Guided bone reconstruction procedures	Guided bone regeneration	19	
	Crestal sinus floor elevation	10	
	GBR + crestal sinus floor elevation	3	
	Socket preservation	39	
	Total		61

Abbreviation: GBR, guided bone regeneration.

The insertion torque ranged between ≥15 and ≤45 Ncm (mean 42.9 ± 4.8 Ncm). Overall, 83.5% of the implants ( *n* = 203) were placed with an insertion torque ranging from ≥35 to ≤45 Ncm. One-hundred-forty-three definitive prostheses were delivered.

One-hundred-sixty-eight implants were rehabilitated with screw-retained prostheses, while the remaining 61 implants received cemented-retained restorations. Moreover, five patients received two-implant-retained overdentures (overall 10 implants), and two patients received hybrid fixed/removable overdentures, completely supported by a CAD/CAM titanium bar, screwed onto four implants (overall eights implants). Data are summarized in Table 6 .

**Table 6:** Definitive restoration distribution

Implant length (mm) and diameter (mm)	Single	FPD	Overdenture a	Hybrid overdenture b	Toronto c	Total
Maxilla	46	9	1	–	7	63
Mandible	58	11	2	2	7	80
Total	104	20	3	2	14	143
Supported implants	1	2 to 3	2	4	4 to 8	243
Screw-retained	71	11	–	–	13	
Cemented-retained	33	9	–	–	1	

Abbreviation: FPD, fixed partial denture.

a Mucosal-supported.

b Implant-supported.

c Fixed full-arch restoration.

At the 1-year follow-up examination, no drop-outs were recorded, but 17 patients (18.9%) with 18 restorations (12.6%) delivered on 34 implants (14%) were lost at the 5-year visit. Two patients died; four patients move to another country/city and refused to return for routine check-up and maintenance, preferring a closer dental clinic; eight patients not able to the visit due to COVID-19 pandemic; and for three patients the reasons were unknown because they did not answer the phone.

**Fig. 6:**
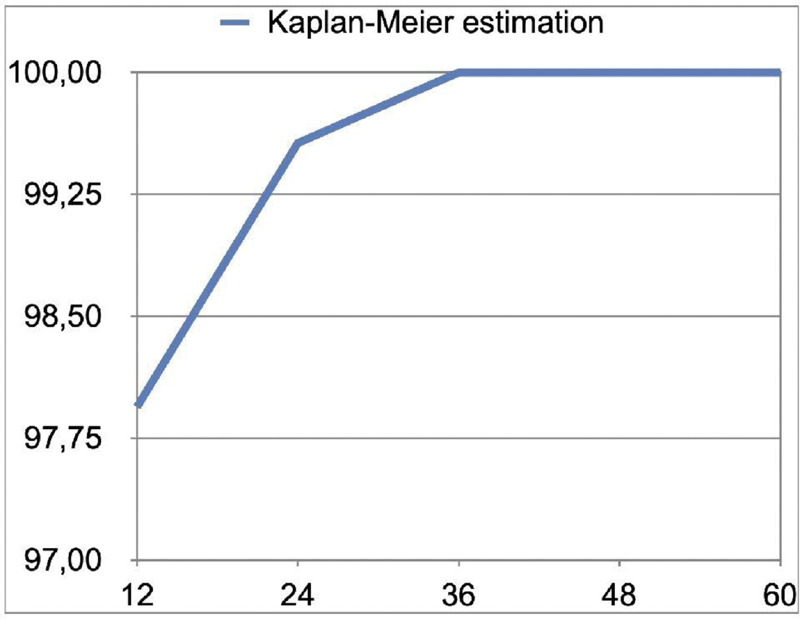
Kaplan–Meier estimation for implant survival.

Overall, at the 5-year examination, six implants failed in six patients, resulting in a cumulative implant survival rate of 97.5%. Five implants failed before definitive loading. One implant failed at the 2-year follow-up. The Kaplan–Meier estimation is reported in Table 7 and Fig. 6 .

**Table 7:** Kaplan–Meier estimation

Follow-up (mo)	Sample at risk (implants)	Drop-outs	Failures	Actual sample	Kaplan–Meier estimation
12	243	0	5	238	97.94
24	230	8	1	229	99.57
36	223	6	0	223	100.00
48	217	6	0	217	100.00
60	203	14	0	203	100.00

No statistically significant differences were found when comparing implant failure within subgroups, except for the insertion torque value. In fact, two failed implants were placed with an insertion torque lower than 35 Ncm (failures 2/7 vs. 4/236; *p* = 0.010). Regarding the other variabilities, two failed implants were placed in combination with bone augmentation procedures ( *p* = 0.6310); one implant was immediately loaded ( *p* = 1.000); two implants were placed immediately after tooth extraction ( *p* = 0.2108). The last failed implant fractured 2 years after definitive prosthesis delivery (0.4%).

At the 5-year follow-up examination, four prostheses failed (2.8%) resulting in a cumulative prosthetic survival rate of 97.2%. One zirconia framework delivered on a complete edentulous patient treated with six implants showed a misfit at the most distal implant, during the try-in session. The framework was remade with no further complications. A second definite restoration made in porcelain fused to a zirconia framework, and delivered on four implants, fractured 5 years after loading. The fractured prosthesis was remade with a new one. Two cemented-retained single crowns delivered to the mandibular molar region failed at the 5-year examination due to abutment damage. Both prostheses were remade with a new screw-retained restoration.

At the 5-year examination, five complications were experienced in the same number of patients (one complication each), resulting in a cumulative prosthetic success rate of 96.5% at the patient level. Three patients with a single screw-retained restoration experienced screw loosening at the 1-year follow-up. The screws were tightened chair-side after prosthesis cleaning, with no further complications except for one patient. For the latter, the patient experienced a new screw loosening at the 2-year follow-up. Occlusion was adjusted, and the screw was replaced, with no further complications. Two patients experienced pain and swelling up to 3 weeks and 4 years after implant placement, respectively, resulting in a MBL greater than 2 mm compared with previous control. Both patients are enrolled in a strictly maintenance program, and no further progressive MBL was experienced.

All the implants were placed at the crestal level or slightly below (0–1 mm, maximum 1.5 mm in case of immediate postextractive implants). At the definitive prosthesis delivery ( *n* = 243), the mean MBL was 0.26 ± 0.25 mm (95% CI: 0.23–0.29). The mean MBL between implant placement and 1 year after loading ( *n* = 243) follow-up was 0.37 ± 0.25 mm (95% CI: 0.33–0.41). The difference was 0.11 ± 0.14 mm (95% CI: 0.09–0.13). Five years after loading ( *n* = 203), the mean MBL for implant placement was 0.41 ± 0.30 mm (95% CI: 0.26–0.34). The difference from the 1-year data was 0.04 ± 0.19 mm (95% CI: 0.01–0.07).

Overall, 4.4% of the implants ( *n* = 9) showed zero MBL, 5 years after loading, while 78.8% of the implants ( *n* = 160) showed a MBL ≥0.1 and ≤0.5 mm. Twenty-five implants (12.3%) showed a MBL ≥0.5 and ≤1.00 mm. Only nine implants (4.4%) showed a MBL greater than 1.0 mm (range: 1.1–2.3 mm). All of these patients were enrolled in a strict hygiene maintenance program. In all of these patients, no surgical procedures were needed. Comparison of MBL and the investigated risk factors was conducted at the 1-year follow-up. 16 It was found a statistically higher MBL for smokers, thin gingival biotype, and GBR. Smokers, thin gingival biotype, and previous GBR were associated with higher MBL. The differences were statistically significant ( *p* < 0.05). 16

The mean ISQ value recorded at implant placement was 71.6 ± 5.5 (minimum: 45; maximum: 88); at the definitive prosthesis delivery (6 months after implant placement), the mean ISQ value was 76.7 ± 4.4 (minimum: 66; maximum: 89). The difference between time points was statistically significant ( *p* = 0.0001).

One hundred and sixty-six implants were placed in bones of type 1 and 2 quality ( *n* = 18). The remaining 77 implants were placed in bones of type 3 and 4.

No statistically significant correlation was found between insertion torque and MBL ( *p* = 0.4216).

## Discussion

The present research was designed as an open-cohort, prospective, case series evaluation, aimed to investigate, over a period of 5 years after definitive restoration delivery, the implant and prosthesis survival and success rates of bone-level titanium implants, featured with a sandblasted/acid-etched surface, and an internal conical connection of 11°, placed in private practice. Furthermore to understand the amount of physiological marginal bone remodeling that could be expected after implant placement and then, in the medium-term follow-up. Finally, to evaluate any complications and possible risk factors, with the aim to prevent complications and failures, including peri-implantitis. The main limitation of the present study was the small sample size, particularly referred to the heterogeneity of the treatments. Unfortunately, the COVID-19 pandemic contributes to a relative higher drop-outs. Nevertheless, at the end of the study, 203 equal implants were placed and patients were followed for at least 5 years after definitive restoration delivery. It is the authors' opinion that 5 years on function could be enough to evaluate the physiological marginal bone remodeling that occurs after biological width establishment, as well as, to understand the trend of annual bone loss.

Six out of a cohort of 243 implants failed during the 5 years after loading examination, scoring a cumulative implant survival rate of 97.5%. These results are completely in agreement with a previous systematic review reporting 5-year follow-up data. Pjetursson and coworkers reported an estimated implant survival rate of 97.2% after 5 years for implants with rough surface. 17 In the present study, five out of six failed implants do not integrate and failed before definitive loading. Kaplan–Meier estimation showed that after an initial risky period (2.06%), the cumulative survival rate becomes higher (100%). A possible explanation was that MBL remains almost stable during the time. At the 5-year follow-up examination, only nine implants (4.4%) showed a MBL between 1.1 and 2.3 mm. On the contrary, 91.1% of the implants showed a MBL ≥0.5 and ≤1.00 mm (of these, 78.8% showed an MBL ≥0.1 and ≤0.5 mm).

According to the preliminary 1-year report, 16 the subgroup analysis demonstrated that previous GBR, thin soft tissue biotype, and smoking habit were associated with statistically significantly higher peri-implant bone loss. These results are in agreement with previous research studies from other authors. Sgolastra and coworkers concluded that smoking habit is associated with higher MBL, implant failure, as well as risk of biological complications, such us peri-implantitis. 18 Moreover, a systematic review with meta-analysis concluded that dental implants placed in patients with initial thick peri-implant soft tissues may expect lower MBL in the short-term period. 19

In the present research, even if GBR is associated with slightly higher MBL, survival rates of implants placed in combination with, or after GBR procedures, were high, without differences when compared with implants placed in native bone. These data are consistent with other reports. 20 21 22 On the other hand, Ramanauskaite and coworkers reported, in a systematic review, lower MBL at the implant inserted into grafted sites, compared with the nongrafted ones. 23 However, Ramanauskaite and coworkers reported a mean difference of approximately 2 mm, compared with the present research, where the difference in MBL was approximately 0.2 mm. Moreover, according to both research studies, implants inserted in previous GBR sites presented a high survival rate.

The one-abutment at one-time protocol and immediate loading have been both proven to reduce the MBL. 24 25 A possible explanation could be that, in the present research, most of the immediately loaded implants were placed flapless, using guided surgery, and they received the definitive abutments on the day of surgery, minimizing the overall peri-implant bone remodeling.

It is well known that primary implant stability is still considered one of the most important criteria for implant success. 26 27 28 29 30 31 In fact, two out of six failed implants had an insertion torque lower than 35 Ncm. Although there is still no consensus that allows us to suggest the ideal insertion torque value to prevent implant complications and failures, it is the authors' opinion that high insertion torque values should be avoided. In the present research, most of the implants (83.5%) reached an insertion torque ranging from 35 to 45 Ncm. According to the manufacturers' protocol, the implant sites were prepared according to the bone density, evaluated at the time of the surgery. Standard implant site preparation was performed in healed sites with a bone density classified as type 2 or 3. 31 Horizontal and/or vertical under-preparation was performed in the case of poor bone quality bone (type 4), sinus lift (with staged implant placement), and postextractive implants. Moreover, in some maxillary cases, osteotomes were used to perform bone spreading, improving bone density and subsequently, primary implant stability.

The major concern of the present 5-year report was the relative higher prosthetic failure and complications. Five years after loading, four prostheses failed and three technical complications were experienced. All of the complications were resolved chair-side, and all the failed prostheses were redone. However, the results of the presented research are in agreement with a previous systematic review. 17 According to Pjetursson and coworkers, the survival rate of metal-ceramic implant-supported fixed dental prostheses was 96.4%, while, in the present research, the absolute value was 97.2%. However, the major difference of the present research is that most of the restorations were metal-free. While dental implants are increasingly becoming the gold standard in replacing missed/failing teeth, the complications associated with them are progressively emerging too. 32 However, one prosthesis failed during the try-in examination. This means that some technical problems could be there during laboratory procedures. The second zirconia framework failed at the 5 years after the loading examination. It is the authors' opinion that zirconia materials were improved during time. Moreover, few years ago, the connection between the prosthesis and the implants was made in zirconia as well. So, today, using improved materials, and titanium connection, it can be expected a longer time free of complications. The last two prostheses were two single crowns. In both cases the hexagon of the abutment broken in five patients after loading. Both implants were wide-diameter implants (6.0 and 7.0 mm) placed in the mandibular molar region. One of these patients was overt bruxer. The second patient was not scheduled as a bruxer, nevertheless, the patient experienced two bereavements (husband and a son) a few months before the prosthetic complication. It is probably that some parafunctional habits appeared. Nevertheless, this point focused the importance of occlusal maintenance besides the normal hygiene maintenance.

Finally, the major clinical contribution from this study was to understand the physiological bone remodeling expected in daily practice, both at the biological wide establishment and yearly. This is of importance to understand risk indicators for peri-implantitis. 33 34 However, it is of great importance to make sure that patients with bleeding on probing and/or plaque index ≥25% were not included in this study. Moreover, all the treated patients were enrolled in an accurate maintenance program with a visit every 4 to 6 months, contributing to lower MBL and incidence or peri-implantitis. 35

One year after loading, the mean MBL was 0.37 mm. This means that implants could be placed at the bone level or slightly below (0.5 mm). In some clinical situations, such as GBR, smoking, and thin soft tissues, the implants should be placed 1 mm below the bone crest. Exceptionally, clinicians can place deep the implants up to 1.5 to 2 mm in case of postextractive implants and very thin biotype. In these cases, one abutment at the one time concept 36 37 or tissue-level implants should be considered.

## Conclusions

Low implant failure and stable peri-implant bone remodeling can be expected using sandblasted/acid-etched conical connection implants in the daily practice, up to 5 years after loading. Previous GBR, smoking habit, and thin soft tissue biotype were the most important variabilities associated with higher MBL. Prosthetic failure and complications may occur. For the latter, property improvements of restorative materials and continuous occlusal controls are needed to reduce these complications.
